# Development of financial performance benchmark of Ministry of Public Health’s hospitals in Thailand

**DOI:** 10.1186/1471-2458-14-S1-O1

**Published:** 2014-01-29

**Authors:** Utoomporn Wongsin

**Affiliations:** 1 Health Insurance System Research Office, Nonthaburi 11000, Thailand

## Background

Continuous monitoring and evaluation of hospital financing performance is necessary for hospital managers and system governance agency, Ministry of Public Health. The objective of this study was to establish a set of financial benchmarks for stakeholders (government agencies, providers and purchasers) for use in hospital financial management.

## Materials and methods

A literature review was performed for selected indicators of benchmarking. This study used secondary data (financial statements) to identify industry standards for hospitals. Data on hospital expenditures of public hospitals were categorised into 5 groups (input mix), which included salary, compensation, drugs, material supply and other expenses. In addition, selected financial ratio analysis (performance) was carried out which included earnings before interest, taxes, depreciation, and amortization (EBITDA), cash ratio, average collection period, payment period and service cost.

## Results

This study found that the salary consumed the highest hospital budget which was followed by drugs. The average salary expense of district hospitals (40%) was higher than that of general hospitals (37%), and regional hospitals (32%), respectively. The drug expense of district hospital was 11%, while it was 19% for general hospitals, and 24% for regional hospitals. In 2011, the median expenditure of district hospitals was 93.72 million Baht, while the expenditures of general hospitals and regional hospitals were 640.10 million Baht and 619 million Baht, respectively. The results of financial ratio analysis showed that district hospitals had lowest liquidity ratio compared to two other types of hospitals. It meant that district hospitals had lowest ability to pay for their short-term debts obligations. Furthermore, they had a problem regarding average payment period ratio. The median of average payment period ratio of district hospital was 400 days, while the median of average payment period ratios of general hospitals and regional hospitals were 223 days, and 186 days, respectively.

## Conclusions

Financial performance benchmarking can give stakeholders a better sense of the components of financial system. This can thus help hospital managers to identify problems within the appropriate time frame and manage them accordingly. The system will enable the mangers to compare their performance with other hospitals of the same level. However, benchmarking financial development needs more reliable information to support and effectively plan for financial management.

